# Treatment of Age-Related Hearing Loss Alters Audiovisual Integration and Resting-State Functional Connectivity: A Randomized Controlled Pilot Trial

**DOI:** 10.1523/ENEURO.0258-21.2021

**Published:** 2021-12-07

**Authors:** Stephanie Rosemann, Anja Gieseler, Maike Tahden, Hans Colonius, Christiane M. Thiel

**Affiliations:** 1 Biological Psychology, Department of Psychology, School of Medicine and Health Sciences, Carl von Ossietzky Universität Oldenburg, Oldenburg 26111, Germany; 2 Cluster of Excellence “Hearing4all,” Carl von Ossietzky Universität Oldenburg, Oldenburg 26111, Germany; 3 Cognitive Psychology, Department of Psychology, School of Medicine and Health Sciences, Carl von Oldenburg 26111 Universität Oldenburg, Oldenburg 26111, Germany

**Keywords:** audiovisual, fMRI, hearing aid, neuroimaging, plasticity, resting state

## Abstract

Untreated age-related hearing loss increases audiovisual integration and impacts resting state functional brain connectivity. Further, there is a relation between crossmodal plasticity and audiovisual integration strength in cochlear implant patients. However, it is currently unclear whether amplification of the auditory input by hearing aids influences audiovisual integration and resting state functional brain connectivity. We conducted a randomized controlled pilot study to investigate how the McGurk illusion, a common measure for audiovisual integration, and resting state functional brain connectivity of the auditory cortex are altered by six-month hearing aid use. Thirty-two older participants with slight-to-moderate, symmetric, age-related hearing loss were allocated to a treatment or waiting control group and measured one week before and six months after hearing aid fitting with functional magnetic resonance imaging. Our results showed a statistical trend for an increased McGurk illusion after six months of hearing aid use. We further demonstrated that an increase in McGurk susceptibility is related to a decreased hearing aid benefit for auditory speech intelligibility in noise. No significant interaction between group and time point was obtained in the whole-brain resting state analysis. However, a region of interest (ROI)-to-ROI analysis indicated that hearing aid use of six months was associated with a decrease in resting state functional connectivity between the auditory cortex and the fusiform gyrus and that this decrease was related to an increase of perceived McGurk illusions. Our study, therefore, suggests that even short-term hearing aid use alters audiovisual integration and functional brain connectivity between auditory and visual cortices.

## Significance Statement

In this study, we showed that first time hearing aid use of six months was related to a decrease in resting state functional connectivity between the auditory cortex and the fusiform gyrus. The decreased connectivity was associated with an increase in perceived McGurk illusions. Further, this increase in McGurk illusions was correlated with decreased hearing aid benefit in auditory speech in noise intelligibility. Our study therefore suggests that hearing aid fitting impacts functional connectivity between auditory and visual regions and audiovisual integration (susceptibility to the McGurk illusion). Further our results suggest, that an increased McGurk susceptibility seems to inhibit the beneficial effect of the hearing aid when tested in auditory only conditions.

## Introduction

One of the most prevalent chronic disorders in older adults is age-related hearing loss (presbycusis), a form of bilateral sensorineural hearing loss that is caused by damage to the cochlea or the auditory nerve and predominantly affects high frequencies. One of the primary treatments for presbycusis is amplification of the auditory input through hearing aids. The use of hearing aids is associated with self-reported improvements in communication because of increased speech clarity (Karawani et al., 2018), improved speech perception, and decreased listening effort (Sarant et al., 2020). Hence, there is a growing interest on the beneficial effects of hearing aid use on brain and cognition in age-related hearing loss (Lin, 2011; Amieva and Ouvrard, 2020). Randomized controlled studies with hearing aid fittings are, however, limited.

Presbycusis is often associated with an increased cross-modal plasticity referring to an increased neural activity in the auditory cortex when processing visual stimuli. Although mainly described in deafness (Lambertz et al., 2005), crossmodal plasticity has also been shown in slight-to-moderate age-related hearing loss (Campbell and Sharma, 2013, 2014). Furthermore, evidence for increased functional coupling of the auditory cortex is provided by research in deaf and cochlear implant patients (Kral et al., 2016; Chen et al., 2017) as well as in hard-of-hearing individuals (Puschmann et al., 2019). Shiell et al. (2015) found a decrease in audiovisual functional connectivity with more years of hearing aid use in early-deaf individuals. Further, a reversal of auditory cross-modal reorganization assessed by cortical visual evoked potentials (Glick and Sharma, 2020) and an increased frontal cortex resting state functional connectivity has recently been identified after six months of hearing aid treatment (Ponticorvo et al., 2021).

Previous studies also revealed that hard-of-hearing participants perceived the McGurk illusion significantly more often than normal-hearing individuals (Stropahl and Debener, 2017; Rosemann and Thiel, 2018; Gieseler et al., 2020). The McGurk illusion refers to an incongruent audiovisual presentation of syllables (auditory ‘ba’ paired with visual ‘ga’) that may lead to the fused percept of a third syllable (‘da’) and is often used to investigate audiovisual integration of speech sounds (McGurk and MacDonald, 1976; MacDonald and McGurk, 1978). Increased McGurk illusion perception in cochlear implant users was related to crossmodal plasticity in the auditory cortex (Stropahl and Debener, 2017). Moreover, increased McGurk illusions were correlated with better audiovisual speech in noise perception (Grant and Seitz, 1998; Gieseler et al., 2020), hence those changes seem to be meaningful for speech comprehension in age-related hearing loss. Thus, although crossmodal plasticity was mostly seen as maladaptive as it might interfere with the clinical benefit of a cochlear implant or a hearing aid (Sandmann et al., 2012), there seem to be adaptive processes aiding in speech perception in cochlear implant patients as well (Stropahl and Debener, 2017).

Increased task-modulated functional connectivity between auditory and visual cortex, when comparing incongruent (McGurk) with congruent audiovisual stimuli, was found in hard-of-hearing compared with normal-hearing participants (Rosemann et al., 2020). Moreover, increased McGurk illusions were correlated with decreased resting state functional connectivity between auditory and motor regions in mild to severe hard-of-hearing individuals (Schulte et al., 2020). Thus, previous research provided evidence for changed audiovisual integration abilities in age-related hearing loss, and functional connectivity alterations in the auditory cortex associated with the McGurk illusion. However, how hearing aid fitting affects, or even reverses, these changes has not been investigated so far.

Therefore, the aim of the current study was to investigate how the McGurk illusion and resting state functional connectivity are altered by hearing aid use in age-related hearing loss. For this purpose, we conducted a pilot randomized controlled hearing aid fitting study in which 16 hard-of-hearing participants were measured one week before and six months after first fitting of a hearing aid (treatment group). The other 16 hard-of-hearing participants were not equipped with a hearing aid and were measured twice at an interval of six months as well (waiting control group). We expected a decrease in the number of McGurk illusions in the treatment compared with the control group (Stropahl and Debener, 2017; Rosemann and Thiel, 2018; Gieseler et al., 2020). Moreover, we hypothesized a decrease in functional coupling of the auditory cortex to visual areas after hearing aid fitting (Shiell et al., 2015; Kral et al., 2016; Chen et al., 2017; Puschmann et al., 2019). We further expected changes in McGurk illusion perception to be related to changes in functional connectivity between auditory cortex and visual as well as motor brain regions (Rosemann et al., 2020; Schulte et al., 2020).

## Materials and Methods

### Participants

We screened *n* = 163 participants between 60 and 80 years of age with self-reported hearing loss and no prior hearing aid experience. Inclusion criteria were a slight-to-moderate, symmetric, age-related hearing loss defined as better-ear pure-tone averages (PTAs) across the frequencies 0.5, 1, 2, and 4 kHz between 18 and 56 dB HL (von Gablenz and Holube, 2015) with PTA differences between left and right ear smaller than 15 dB HL and air-bone gaps no larger than 10 dB HL. In addition, participants had to be right-handed, German native speakers, have normal or corrected-to-normal vision and no current or previous self-reported neurologic or psychiatric disorders, including tinnitus. Furthermore, participants were screened for dementia and excluded in case of suspected dementia (age normed DemTect scores of >8; Kalbe et al., 2004). Seventy-one participants were initially included in the study, of whom *n* = 58 completed behavioral testing. Subjects with no MRI contraindications were invited for an additional MRI scan. In this paper, we report data of *n* = 32 participants with complete behavioral and MRI data. Of those, *n* = 16 participants were allocated to the treatment group and *n* = 16 to the waiting control group.

Approval for the study was obtained from the local ethics committee of the University of Oldenburg (no. 25/2018), and the study was conducted in accordance with the Declaration of Helsinki as well as the European Union General Data Protection Regulation. All subjects signed a written informed consent form and were paid for participation.

### Treatment and control group

Treatment and control group were stratified with respect to hearing impairment (PTA), age and cognitive performance (as assessed by the dementia screening test DemTect; Kalbe et al., 2004). The mean age and hearing impairment of the participants at screening was 71.2 ± 5.1 years and 28.5 ± 9.6 dB HL (range 20.0–56.3 dB HL) in the treatment group and 70.0 ± 4.7 years and 27.2 ± 7.5 dB HL (range 17.5–45.0 dB HL) in the control group, respectively.

Participants in the treatment group were fitted with bilateral Signia Pure 7Nx/px hearing aids with M-receivers and click dome (semi-) open coupling by trained acousticians at the Hörzentrum Oldenburg GmbH. For the fitting formula, NAL-NL2 with fixed pinna-preserving Omni-Mode and default noise reduction was set as default. For all fittings, *in situ* measurements were done. If the participants were uncomfortable with the default setting, the acousticians allowed for little fine-tuning to avoid drop-outs (as much as needed, as little as possible) starting with the simplest approach by (1) trying to master gain, (2) frequency-dependent gain, (3) compression, and (4) allowing for additional noisy-environment program only if the subject explicitly required it. All changes were documented. The participants were instructed to wear their hearing aids daily for at least 6 h and gave their written consent to log the data of the hearing aid. The waiting control group was offered a hearing aid fitting by the Hörzentrum Oldenburg GmbH after the study was completed.

### Experimental procedure

All participants underwent behavioral and MRI measurements one week prior and six months after potential hearing aid fitting. Participants in the treatment group were additionally assessed one month after the fitting (data not reported).

**Figure 1.**
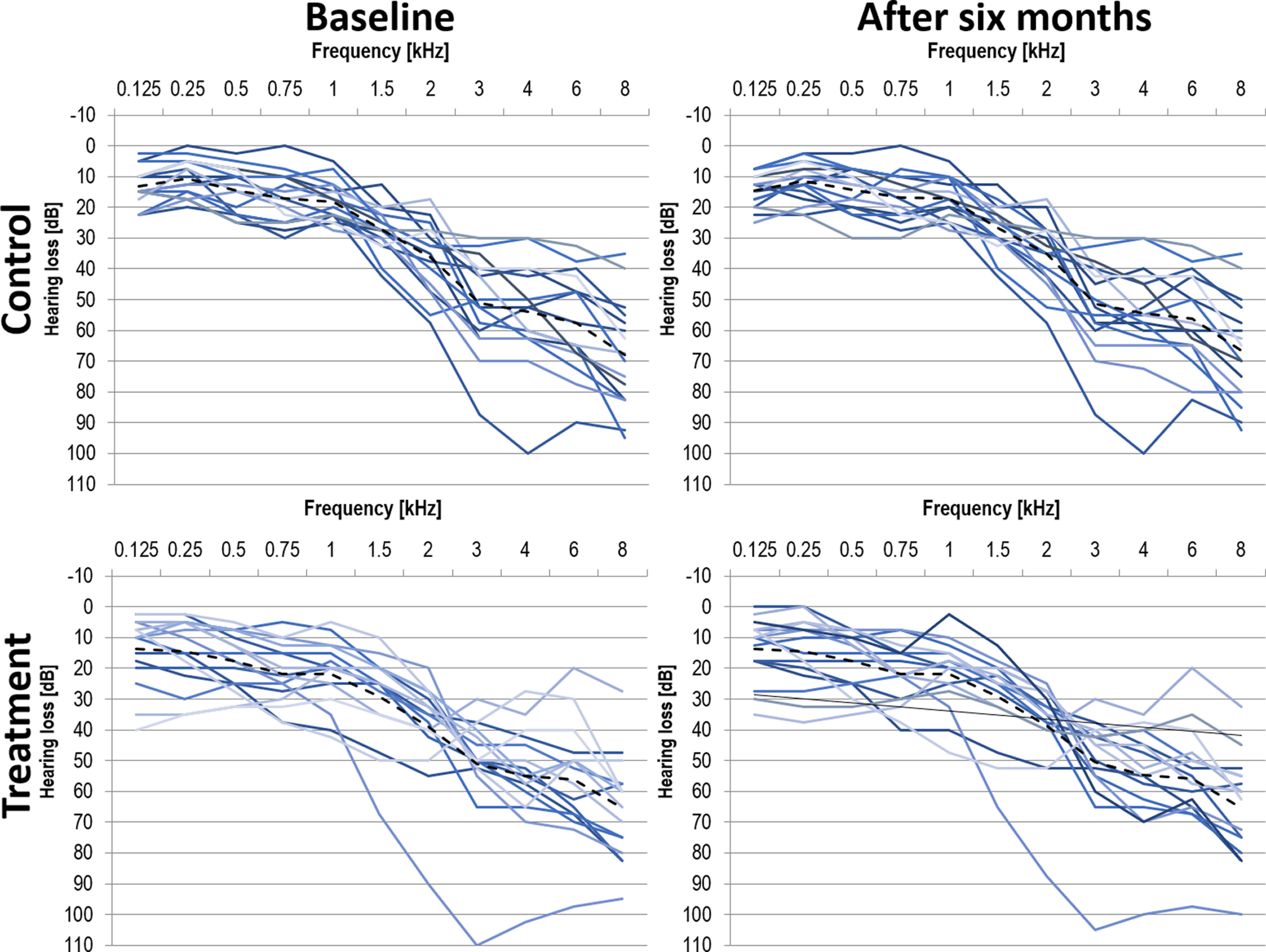
Individual pure-tone audiograms for participants in the control group (top panels) and treatment group (bottom panels) at baseline (left panels) and after six months (right panels). Mean across both ears as well as group averages (dashed line).

The individual pure-tone audiograms for the baseline and six-month postassessment are shown in Figure 1. Note that there were no changes in the PTA for neither the control nor the treatment group after six months.

### Behavioral measurements

Behavioral measurements included a pure-tone audiometry, cognitive screening [Montreal Cognitive Assessment (MOCA), Nasreddine et al., 2005; DemTect, Kalbe et al., 2004] and audiovisual tasks (McGurk task, sound-induced flash illusion, audiovisual speech in noise intelligibility task described in Llorach et al., 2021). We focused only on the McGurk task, the audiovisual speech in noise intelligibility task and resting state measurements.

Pure tone audiometry was performed in a soundproof chamber using air conduction pure-tone audiometry for the frequencies 125, 250, 500, 1000, 2000, 4000, 6000, and 8000 Hz.

The McGurk task was performed in a soundproof chamber, ∼60 cm in front of the screen. The stimuli were presented on a 17’’ Iiyama ProLite T 1731 SR-B1 monitor (spatial resolution: 1280 × 1024 pixels; luminance: 200 cd/m^2^; refresh rate: 60 Hz) and via Sennheiser HDA200 headphones delivered by the USB audio interface RME Fireface UC (http://www.rme-audio.de/en/products/fireface_uc.php). Stimulus presentation was controlled by Presentation software (version 18.2, Neurobehavioral Systems). The task consisted of typical McGurk syllables that were presented as either auditory-only, visual-only, audiovisual congruent, or audiovisual incongruent conditions (the McGurk illusion). We used the syllables ‘ga’ and ‘ba,’ which commonly lead to the fused percept ‘da’ when presented as incongruent stimuli (visual ‘ga’ with auditory ‘ba’). These stimuli were a subset of syllables that were recorded in the Department of Media Production at the University of Oldenburg and consisted of video and audio files of a male speaker articulating the different syllables in front of a dark gray background (Rosemann and Thiel, 2018). The syllables ‘ga’ and ‘ba’ were presented in either auditory-only or visual-only as well as audiovisual congruent conditions in addition to the incongruent presentation of the McGurk illusion condition. The audiovisual conditions (both congruent and incongruent) were presented either synchronously [stimulus onset asynchrony (SOA) of 0 ms] or asynchronously (SOAs of 70–420 ms in steps of 50 ms). The different SOAs were used to assess the temporal domain in which the McGurk illusion is most likely perceived. In these asynchronous presentations, the auditory stimulus followed the visual stimulus. Each SOA was repeated ten times and unimodal conditions were presented five times. Each trial started with a 1000-ms blank screen followed by a jittering 600- to 800-ms black fixation cross on a white background. Then, the video/audio files were presented. Last, the response display with the syllables (‘ba,’ ‘ga,’ ‘da’) as response options (labeled as 1, 2, 3; three-alternative forced choice) was presented. Participants were instructed to report what they heard. The response (respective number from the response display) was given orally to the experimenter and the next trial only started after the experimenter had entered the response. The total duration of the McGurk task was 20 min. The auditory stimuli in the McGurk task were presented via headphones and the loudness was adjusted individually for each participant before the test and training session (in steps of 1 dB to medium loudness starting at 65 dB SPL). The mean medium loudness level of the auditory stimuli was 68 dB for the baseline measurement and 69 dB for the six months re-test measurement in the control group and 69 and 71 dB in the treatment group, respectively. Importantly, the participants of the treatment group were measured unaided, i.e., without their hearing aids, at the re-test assessment to assess potential general changes that are not associated with specific hearing aid settings.

The auditory and audiovisual speech intelligibility tasks were the Oldenburg Sentence test (Oldenburger Satztest, OLSA; Wagener et al., 1999a,b,c) and the corresponding audiovisual version of it (please find a detailed description in Llorach et al., 2021). The OLSA consists of five-word sentences and the task for the participants is to repeat what they heard. Each testing condition involved 20 sentences each in fluctuating background noise (ICRA4-250). The presentation of the conditions was randomized (auditory only and audiovisual conditions as well as aided and unaided conditions after six months in the treatment group). Before the testing, each participant conducted a training of ten sentences in each condition. For each condition, the corresponding 80%-speech reception thresholds were determined, referring to the signal-to-noise ratio that yields 80% intelligibility based on performance (word scoring). An adaptive procedure was applied (fixed background noise presentation level at 65 dB SPL; the target speech was adaptively varied based on the previous answer, starting at 0 dB SNR).

### MRI measurements

The MRI measurements took place on a separate day in close proximity to the behavioral assessment. Imaging data were acquired on a 3T whole-body Siemens Magnetom Prisma MRI machine with a 20-channel head coil. Resting state fMRI data were recorded with an ascending echo planar imaging sequence (320 T2*-weighted volumes, TR = 1500 ms, TE = 30 ms, voxel size = 2.2 × 2.2 × 3 mm, 25 slices). Participants had to fixate a white fixation dot that was presented on a black screen. The fixation dot was presented with the Presentation software (version 18.3, Neurobehavioral Systems) via a projector behind the bore of the MRI (DATAPixx2, VPixx Technologies Inc.). Anatomical images were acquired with a 3-D T1-weighted sequence (MP-RAGE, TR = 2000, TE = 2.07, flip angle = 9°, voxel size = 0.75 mm, field of view = 240 × 240, 224 sagittal slices).

### Analysis of behavioral data

To assess general task performance in the McGurk task, we computed mean performance measures (% correct) for audiovisual congruent conditions (across all SOAs), auditory-only and visual-only conditions for each participant. Potential differences in general task performance between groups at baseline and after six months were assessed by repeated-measures general linear model (GLM) on the percent of correctly identified syllables with condition (audiovisual congruent/auditory/visual) as within-subject factor and group (treatment/control) as between-subject factor.

To assess audiovisual integration, the incongruent McGurk (illusion) trials underwent a detailed analysis to investigate potential group differences in the susceptibility to the McGurk illusion (defined as fusion reports ‘da’). Since the response options in the incongruent condition can be either the auditorily-presented syllable (‘ba’), the visually-presented syllable (‘ga’) or the fused illusion percept (‘da’), a first analysis focused on the percent for each response option across SOAs using a repeated-measures GLM with group as between-subject and response as within-subject factors (for baseline and after six months). A second analysis focused on the fused percept as a function of SOA using a repeated-measures GLM with group as between-subject and SOAs within-subject factors (for baseline and after six months).

Two *post hoc* correlation analyses were performed to investigate (1) whether the McGurk susceptibility is a valid measure of audiovisual integration (correlation of McGurk illusions with audiovisual speech intelligibility) and (2) whether the change in the McGurk susceptibility is a positive or negative outcome for speech intelligibility (correlation of the change in McGurk susceptibility between baseline and retest assessment with benefit of aided vs unaided speech intelligibility).

### Analysis of MRI data

Resting state functional connectivity data were analyzed with the Statistical Parametric Mapping software package (SPM12, Wellcome Department of Imaging Neuroscience, London, United Kingdom) based on MATLAB 2016b and the CONN toolbox (Whitfield-Gabrieli and Nieto-Castanon, 2012). Images were preprocessed in SPM including spatial realignment estimation, slice timing correction and coregistration. Next, a normalization to the Montreal Neurologic Institute space using parameters obtained from segmentation of the anatomic T1-weighted image and spatial smoothing using a Gaussian kernel with a full width at half maximum of 8 mm was applied. After normalization, data processing proceeded in CONN: remaining physiological and movement artefacts were removed by linear regression. The BOLD signal from white matter and cerebrospinal fluid as well as realignment parameters were used for denoising. Subsequently, a bandpass filter (0.008–0.9 Hz) and linear detrending was applied. First-level analyses revealed Fisher-transformed correlation coefficients for each subject. Individual connectivity maps were subsequently entered into two second-level analyses to identify treatment-dependent changes in resting state connectivity over time. The first analysis was a whole-brain analysis with seed in the auditory cortex (seed-to-voxel analysis), the second analysis was a region of interest (ROI) analysis (ROI-to-ROI) focusing on connectivity changes between (1) auditory cortex and fusiform face area and (2) the auditory cortex and M1 lip area.

The main seed in this study was positioned in the left and right Brodmann areas 41 and 42 (defined using the Automated Anatomical Labeling (AAL) ROI-Library within the WFU Pickatlas). This pooled seed was used for the whole-brain analysis and for the ROI-to-ROI analysis. The fusiform gyrus was used as a ROI because of its involvement in face processing (Kanwisher et al., 1997; Kanwisher and Yovel, 2006) and its neural activity during the presentation of McGurk syllables (Rosemann et al., 2020). Further, coupling between auditory and visual areas was shown to be increased in deafness and hearing impairment (Shiell et al., 2015; Kral et al., 2016; Chen et al., 2017; Puschmann et al., 2019). We further used the M1 lip area as a ROI because its neural activity was shown to be related to the McGurk illusion in healthy volunteers (Murakami et al., 2018). Further, studies in age-related hearing loss demonstrated increased neural activity in motor cortex but decreased coupling between auditory and motor regions with increased McGurk illusion responses (Rosemann et al., 2020; Schulte et al., 2020). The ROI in the left and right fusiform gyrus was provided by the atlas implemented in CONN. The ROI in the left M1 lip area constitutes a sphere of 8-mm radius around the coordinates (*x* = −44, *y* = −11, *z* = 34) provided by Murakami et al. (2018).

In order to assess changes in functional resting state connectivity of the auditory cortex to the rest of the brain after hearing aid fitting, we computed an interaction between group (treatment/control) × measurement time point (baseline/after six months) with a height threshold of *p* < 0.001 and a cluster corrected threshold of *p*FWE < 0.05. For the ROI analysis, connectivity values between (1) the auditory cortex and the fusiform gyrus as well as (2) the auditory cortex and the M1 lip area (ROI-to-ROI analyses) were extracted. Changes in connectivity values from auditory to fusiform and the M1 lip area between measurement time points were compared across groups (using repeated-measures ANOVAs) and correlated with changes in the McGurk illusion (Pearson correlation). These analyses were computed in JASP (JASP Team, 2020; version 0.14.1).

## Results

### Hearing aid use

Although all participants were instructed to wear the hearing aid for at least 6 h/d, the average daily wearing period ranged from 1 to 12 h, with a median of 7 h. Given the small number of subjects we refrained from excluding three subjects who had been wearing their hearing aids for <5 h/d as none of them had been detected as an outlier during the analysis.

**Table 1:** Mean values (±standard deviation) for performance in the McGurk task

Condition	Measurement	Control group	Treatment group
Audiovisual congruent trials: % correct	Baseline	87.9 ± 21.7	93.1 ± 15.4
	After 6 months	90.9 ± 16.6	97.9 ± 3.3
Auditory-only trials: % correct	Baseline	84.4 ± 18.6	85.6 ± 19.3
	After 6 months	93.1 ± 14.5	86.0 ± 13.5
Visual-only trials: % correct	Baseline	65.0 ± 22.8	70.0 ± 15.5
	After 6 months	71.9 ± 19.1	68.7 ± 15.1

All conditions except the incongruent McGurk illusion condition are presented for the baseline as well as re-test (after six months) measurements.

### Behavioral results

**Figure 2.**
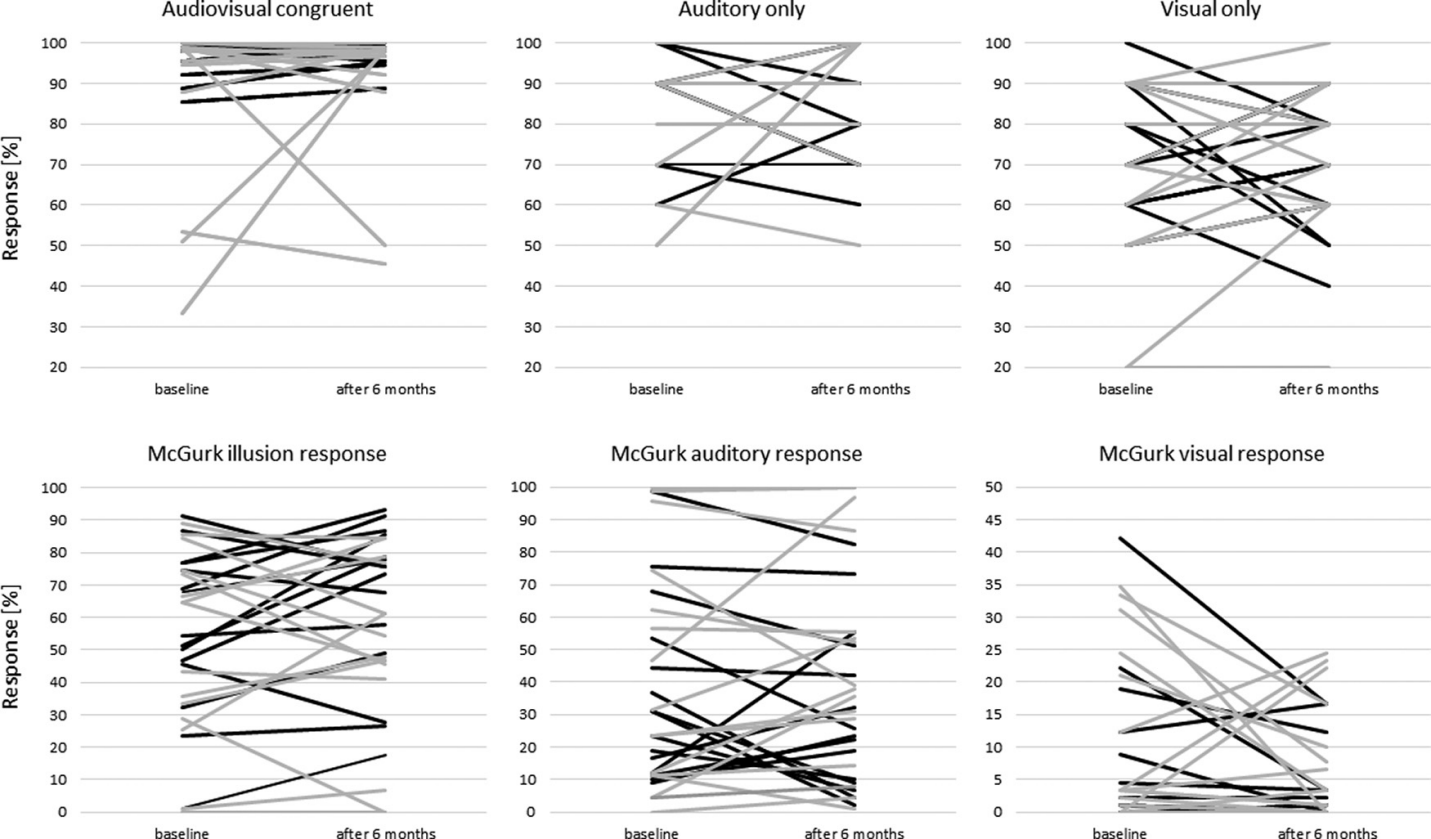
Individual McGurk data. Correct responses (%) shown for the audiovisual congruent, auditory only and visual only conditions in the top panel (as in Table 1). Different responses (%) given in the McGurk illusion trials are shown in the lower panel (as in Table 2). The responses of subjects in the treatment group are shown in black and those in the control group in gray (mean values for each participant).

The mean performance values for both groups and measurements for audiovisual congruent, auditory-only, and visual-only conditions are presented in Table 1. Individual data for all participants is shown in Figure 2, upper panel. The repeated-measures GLM revealed a significant main effect of condition at both baseline and re-test assessment after six months (baseline: *F*_(2,60)_ = 34.49, *p*_adj_ < 0.001, GG_eps_ = 0.76, η^2^*p* = 0.54; after six months: *F*_(2,60)_ = 44.75, *p*_adj_ < 0.001, GG_eps_ = 0.78, η^2^*p* = 0.61), whereas neither the main effect of group nor the group × condition interaction yielded significance. Hence, general task performance did not differ between the treatment and control group for neither baseline nor re-test assessment. *Post hoc* comparisons showed that both groups performed significantly better in both the auditory-only and audiovisual congruent condition than in the visual-only condition (all *p*_adj_ < 0.001).

**Table 2:** Mean values (±standard deviation) for performance in the McGurk audiovisual incongruent trials

Condition	Measurement	Control group	Treatment group
Audiovisual incongruent trials: illusion percept (‘da’)	Baseline	48.1 ± 31.3	54.5 ± 25.1
	Post 6 months	46.0 ± 29.9	65.6 ± 24.5
Audiovisual incongruent trials: auditory percept (‘ba’)	Baseline	41.0 ± 35.9	36.9 ± 26.5
	Post 6 months	46.5 ± 33.8	30.6 ± 25.2
Audiovisual incongruent trials: visual percept (‘ga’)	Baseline	10.9 ± 13.3	8.6 ± 12.4
	Post 6 months	7.5 ± 9.1	3.8 ± 6.1

Percent values are shown for the three response options for the baseline as well as re-test (after six months) measurements (mean across all SOAs).

**Figure 3.**
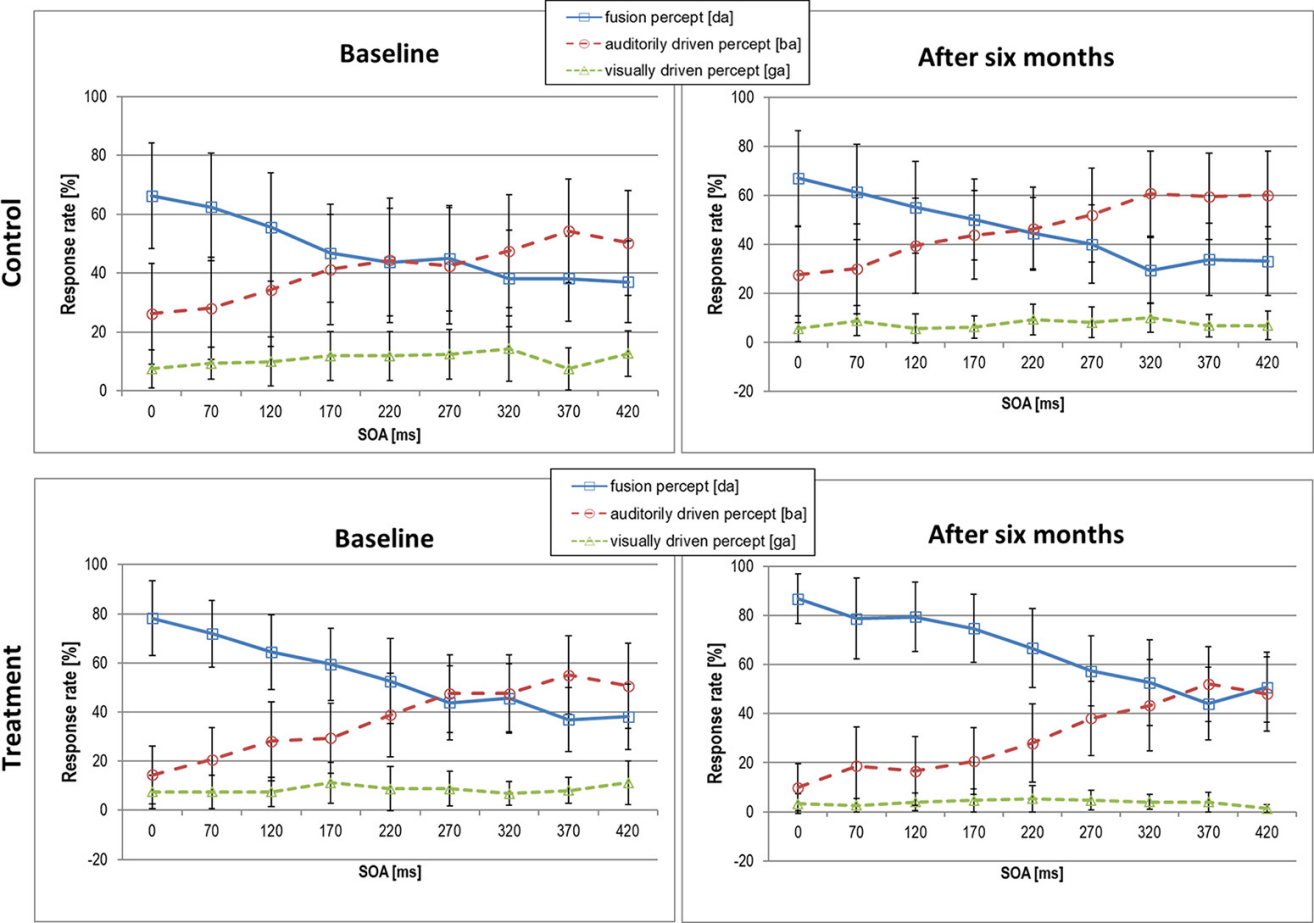
Response rate across SOAs in the audiovisual incongruent (illusion) condition of the McGurk task. Data shown for the control (top panel) and treatment group (bottom panel) for the baseline assessment (right panels) and assessment after six months (left panels; mean values ± standard deviation).

To assess changes in audiovisual integration we analyzed the amount of McGurk illusions (‘da’ response), auditory (‘ba’), and visual (‘ga’) percepts in incongruent trials for each group at both measurement time points (response mean across SOAs shown in Table 2; individual data are shown in Fig. 2; responses as a function of SOA shown in Fig. 3). At baseline, the expected significant main effect of response was not accompanied by significant group, nor by significant response × group interaction effect. Across all SOAs, both groups perceived the audiovisual illusion and the auditory percept more often than the visual percept. Note, however, the large variability in both groups.

After six months, we again found a main effect of response together with a significant trend for a response × group interaction with a medium effect size (response: *F*_(2,60)_ = 23.47, *p*_adj_ < 0.001, GG_eps_ = 0.55, η^2^*p* = 0.45; response × group: *F*_(2,60)_ = 2.96, *p*_adj_ = 0.055, GG_eps_ = 0.55, η^2^*p* = 0.092). As presented in Table 2 and visualized in Figure 2, the control group perceived the audiovisual illusion after six months almost equally often as in the baseline assessment, whereas the treatment group showed an increase in McGurk illusions after six months of hearing aid use.

The correlation between responses given at baseline and responses given after six months for all participants was significantly correlated for all three response options, while the correlation was stronger for the McGurk illusion (*r* = 0.797, *p* < 0.001) and the auditory response (*r* = 0.778, *p* < 0.001) than for the visual response (*r* = 0.366, *p* = 0.043).

To assess potential group differences and changes in the McGurk illusion as a function of SOA, we first looked at the baseline assessment. As expected, the main effect of SOA was significant with decreasing illusion reports with increasing SOAs (*F*_(8,240)_ = 17.19, *p*_adj_ < 0.001, GG_eps_ = 0.52 η^2^*p* = 0.36), whereas neither the main effect of group nor the SOA × group interaction were significant. Hence, although the treatment group showed slightly higher values in the amount of McGurk illusions at baseline, those differences were not statistically significant.

At the re-test assessment, we found a significant main effect of SOA together with a significant trend for a main group effect with a medium effect size (SOA: *F*_(8,240)_ = 18.07, *p*_adj_ < 0.001, GG_eps_ = 0.29, η^2^*p* = 0.38; group: *F*_(1,30)_ = 3.97, *p* = 0.05, η^2^*p* = 0.12). The treatment group showed more McGurk illusions than the untreated peers.

Figure 3 shows how the amount of fusion percept decreases with increasing SOAs, i.e., the further the onsets of the lip movement and the tone are apart, the less likely they are integrated. Yet, it is not until 220 ms for the control group and 270 ms for the treatment group that the auditory percept is perceived more often than the fused percept. After six months, the treatment group shows an even more pronounced integration of visual and auditory information up to an SOAs of 320 ms. Importantly, in the treatment group, the performance in the McGurk task across the different conditions did not correlate with the mean daily wearing period of the hearing aid.

Two *post hoc* correlation analyses were performed to investigate (1) whether the McGurk susceptibility is a valid measure of audiovisual integration and (2) whether the change in the McGurk susceptibility is a positive or negative outcome for speech intelligibility. The results for the speech intelligibility task can be seen in Table 3. The treatment group was tested in an aided and unaided condition after six months. No significant differences between the two groups were obtained in the unaided measurement conditions (*p* > 0.1).

**Table 3:** Mean values (±standard deviation) for performance in speech intelligibility task in auditory (A only) and audiovisual (AV) conditions

Condition	Measurement	Control group	Treatment group
A only unaided	Baseline	−3.69 ± 5.48	−3.98 ± 6.06
	Post 6 months	−7.70 ± 3.18	−7.54 ± 3.98
AV unaided	Baseline	−10.65 ± 5.03	−10.74 ± 6.19
	Post 6 months	−14.42 ± 3.75	−14.29 ± 3.75
A only aided	Post 6 months	–	−9.21 ± 3.83
AV aided	Post 6 months	–	−15.48 ± 5.49

The treatment group was tested in aided and unaided conditions in the re-test measurement after six months (SNR at 80% speech intelligibility).

**Figure 4.**
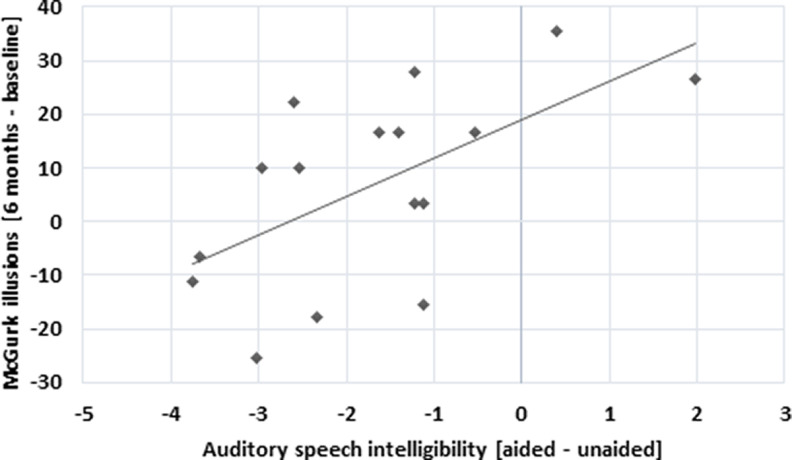
Positive correlation between the difference in McGurk illusions (six months – baseline) and auditory speech intelligibility test conditions (aided – unaided) in the treatment group.

The correlational analysis between audiovisual speech intelligibility values and McGurk susceptibility at baseline showed a correlation at trend level (Pearson’s *r* = −0.457, *p* = 0.075), indicating better audiovisual speech intelligibility with higher number of McGurk illusions. The same correlation was not significant after six months (unaided condition, all participants). As a next step, we computed a correlational analysis for the treatment group only, evaluating the benefit of aided versus unaided measurement conditions and the change in McGurk susceptibility over time. While this analysis showed a positive correlation for the auditory speech intelligibility (Pearson’s *r* = 0.6, *p* = 0.014; Fig. 4), this was not significant for the audiovisual speech intelligibility.

### Neuroimaging results

**Figure 5.**
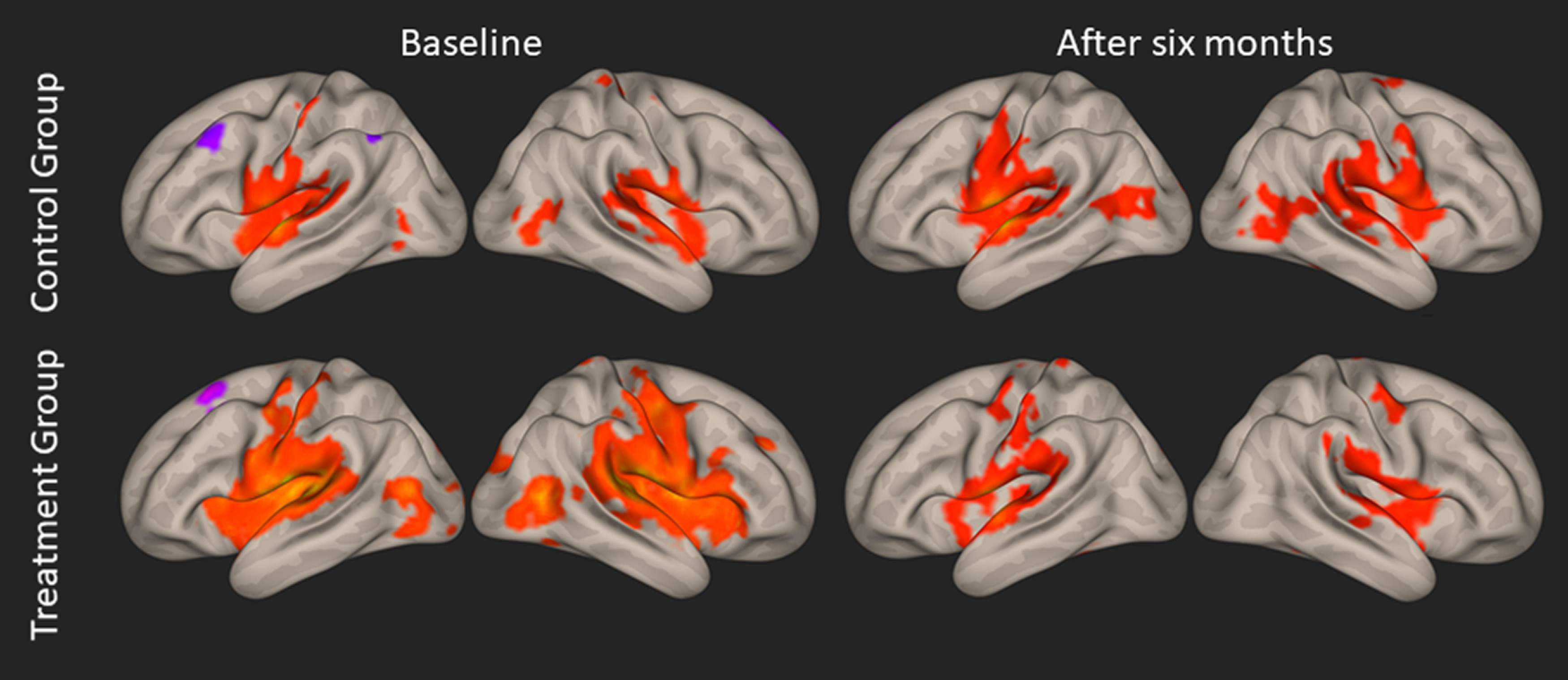
Whole-brain resting-state functional connectivity of the left and right auditory cortex (for each measurement and each group). Left and right hemispheres are presented on left and right side, respectively (*p* < 0.05; FWE corrected on the cluster level). Positive correlations are shown in red, negative correlations are shown in purple.

We conducted a functional connectivity analysis of the auditory cortex (left and right Brodmann areas 41 and 42) to the whole brain at baseline and after six months (see Fig. 5). The auditory resting state network comprised areas in the occipital, temporal and parietal lobe as well as the cingulate cortex and parts of the frontal cortex. Mostly, a positive correlation between neural activity in these regions was obtained, however coupling with frontal areas (superior, middle and orbito- frontal gyrus) and parts of the left parietal cortex (inferior parietal lobule) was negative. To assess the changes in functional resting state coupling with hearing aid fitting, we computed the interaction between group (treatment/control) × measurement time point (baseline/after six months). This analysis did not reveal any significant results (even when the threshold was lowered to *p* < 0.001, uncorrected for multiple comparisons).

**Figure 6.**
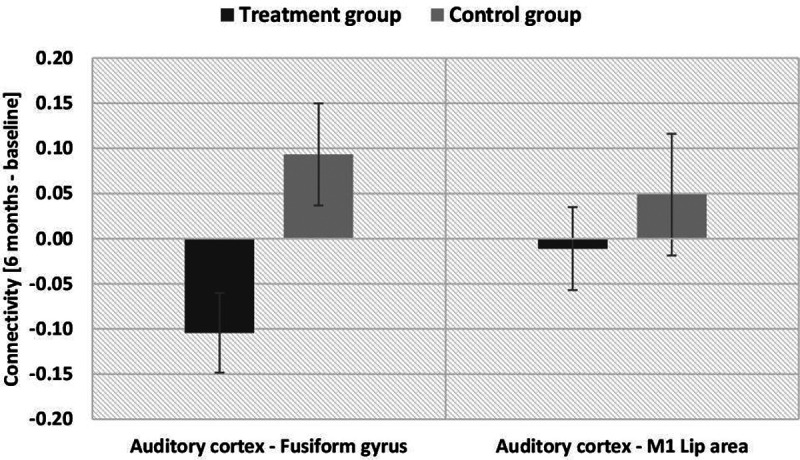
Change in resting-state functional connectivity between measurements at baseline and after six months for the two groups (ROI-to-ROI analysis). Negative values refer to a decrease in connectivity between the respective regions after six months, positive values refer to an increase in connectivity after six months; ROIs were positioned in (1) left and right auditory cortex, (2) left and right fusiform gyrus, (3) left M1 lip area (mean values with standard error of the mean).

In the second step, we implemented a ROI analysis and extracted functional connectivity values between the ROIs in the auditory cortex (left and right Brodmann areas 41 and 42) and the fusiform gyrus as well as between the auditory cortex and the M1 lip area (ROI-to-ROI analysis) for each group and measurement time point (baseline and after six months). The interaction between group and measurement time point with respect to functional connectivity of the auditory cortex and the fusiform gyrus was significant (*F*_(1,30)_ = 7.566, *p* = 0.01, η^2^ = 0.142; Fig. 6, left). The control group showed a numeric increase coupling between auditory cortex and fusiform gyrus after six months (which was however not significant), while the treatment group showed a significant decrease in coupling between those areas. We did not obtain a significant interaction between group and measurement time point in the resting state connectivity of the auditory cortex and M1 lip area (*F*_(1,30)_ = 0.538, *p* = 0.469, η^2^ = 0.007; Fig. 6, right).

**Figure 7.**
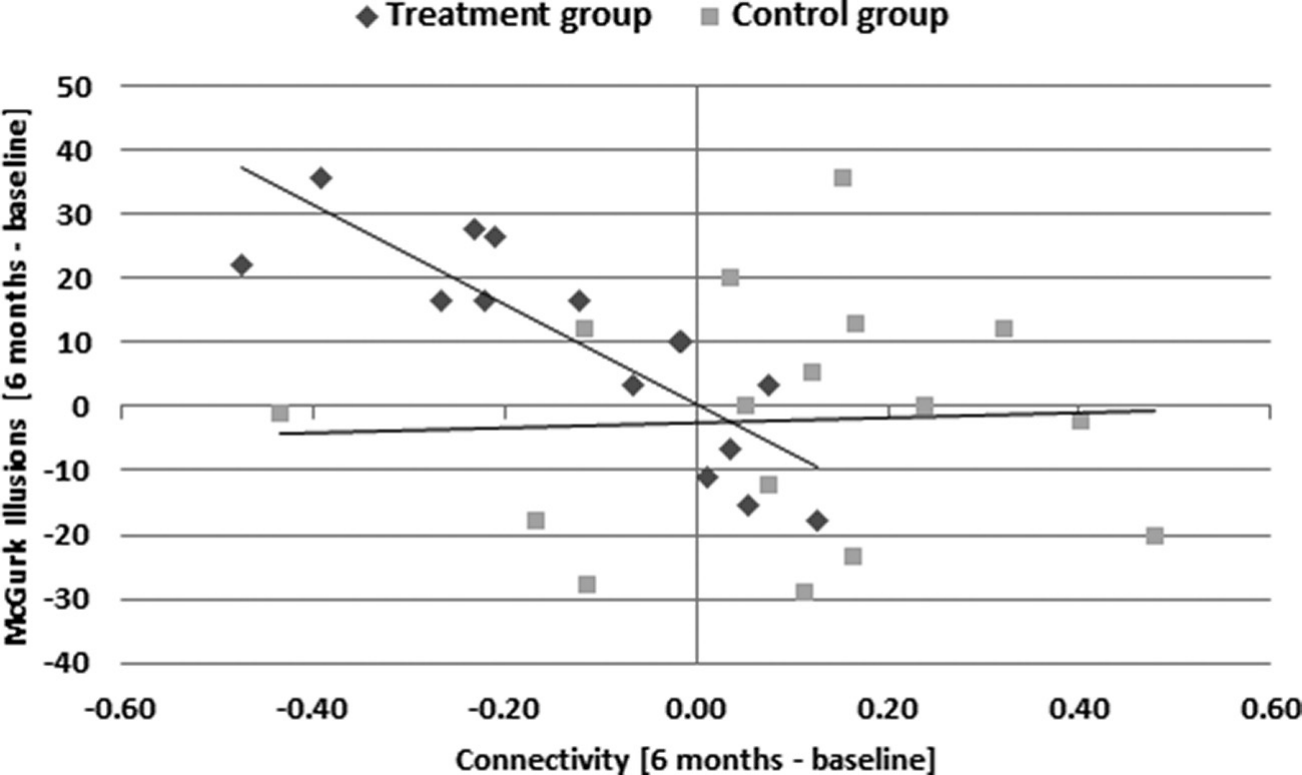
The relation between the change in resting-state functional connectivity between auditory cortex and fusiform gyrus and the change in percentage of perceived McGurk illusions (six months – baseline measurement). Data were pooled over ROIs in left and right hemispheres.

Importantly, this decrease in resting state functional connectivity between the auditory cortex and fusiform gyrus was correlated with an increase in reported fused McGurk percepts in the treatment group (Pearson’s *r* = −0.846, *p* < 0.001), but not in the control group (Pearson’s *r* = 0.045, *p* = 0.869; Fig. 7). Hence, the increase in audiovisual integration after hearing aid fitting cooccurred with a reduction of functional brain connectivity between auditory and visual brain regions. The average daily hearing aid use was not correlated with any of the connectivity measures obtained in the resting-state functional connectivity analysis.

## Discussion

This pilot randomized controlled study investigated the effect of hearing aid fitting on audiovisual integration abilities in slight-to-moderate age-related hearing loss. For this aim, one group of hard-of-hearing participants was measured one week before and six months after fitting and use of hearing aids. The waiting control group was measured twice with a duration of six months between measurements and was not equipped with a hearing aid during that time. Our results provide evidence that six months of hearing aid use is associated with a decrease in functional connectivity between the auditory cortex and fusiform gyrus that is correlated with an increase in perceived McGurk illusions. Furthermore, the increase in perceived McGurk illusions was correlated with a decreased hearing aid benefit in auditory speech intelligibility.

### The effect of hearing aid use on the McGurk illusion

Audiovisual integration abilities in hard-of-hearing participants were assessed with the McGurk illusion. We hypothesized a decrease in the number of perceived McGurk illusions in the treatment group compared with the control group after six months, arguing that regular hearing aid use might lead to a bias toward the auditory percept, as in normal-hearing participants, rather than the integration of incongruent audiovisual information (Stropahl and Debener, 2017; Rosemann and Thiel, 2018; Gieseler et al., 2020). Our results demonstrated a positive trend of hearing aid use on the McGurk illusion. However, in contrast to our hypothesis, the treatment group showed an increase in perceiving the McGurk illusion over time when compared with the control group.

Previous research using the McGurk illusion in untreated, hard-of-hearing individuals has demonstrated that they perceive the McGurk illusion more often than normal-hearing individuals (Stropahl and Debener, 2017; Rosemann and Thiel, 2018; Gieseler et al., 2020). In line with those studies, our results of the baseline assessment also showed a relatively high amount of McGurk illusions in relation to the auditory percept in both our hard-of-hearing groups. Furthermore, we extended previous results by varying the stimulus-onset asynchronies between the visual information (lip movement) and auditory information (tone). We found that the participants of the control and treatment groups integrated the incongruent information up to SOAs of 220 and 270 ms, respectively, at the baseline assessment. After six months, the treatment group reported more McGurk illusions than their untreated peers, even up to temporal disparities of 320 ms. The effect was, however, only significant at trend level. The results further showed, that along with an increased McGurk illusion perception, the treatment group showed a 50% reduction in selecting the visual input during McGurk trials, and only a reduction of 20% in selecting the auditory input. This suggests that answers were less likely given for the visual input after six months of wearing a hearing aid. Surprising was however that the McGurk illusion was still perceived more often and the auditory input has been selected fewer than at the baseline assessment. It therefore seems that after hearing aid use of six months, the auditory modality is not yet regarded as more reliable than before using the hearing aid. Consequently, no increase in perceiving the auditory syllable, and thus no decrease in McGurk illusion perception, was observed. A similar longitudinal study from Glick and Sharma (2020) also found no change in benefit from visual cues in a speech-in-noise task after six months of hearing aid use. However, the hard-of-hearing group did not significantly benefit from visual cues compared with a normal-hearing control group at baseline, which probably explains the absent change of visual benefit because of wearing a hearing aid. It is possible that a longer period of hearing aid use may cause the auditory input to be perceived as the more reliable modality than the visual input, which may also result in a decrease in the number of perceived McGurk illusions. Another explanation might be that the individuals in the treatment group were measured without their hearing aids which created a rather unusual and difficult listening situation in which the auditory input was not perceived as reliable as with the hearing aids.

On a different note, it should be mentioned, that previous research showed rather large variability in the susceptibility of the McGurk illusion (Mallick et al., 2015; Magnotti and Beauchamp, 2018). Differences in susceptibility were attributed to age, gender, culture or native language (Magnotti and Beauchamp, 2018) but also lip-reading abilities (Strand et al., 2014; Brown et al., 2018). Similarly, high variation for fusion effects have been reported for other audiovisual paradigms as for instance the sound-flash illusion (de Haas et al., 2012). Further, unlike other paradigms (such as the sound-flash illusion), the McGurk effect uses syllables as stimuli, which constitute a rather naturalistic setting closer to real world scenarios of audiovisual speech perception. Importantly, the test-re-test correlation in the McGurk susceptibility with a duration of one year between measurements was found to be high (*r* = 0.91) in a sample of 165 participants (Mallick et al., 2015). Although there is a large inter-subject variability in the susceptibility to the McGurk illusion, the McGurk effect is frequently used and probably the most popular paradigm to investigate audiovisual integration (Tiippana, 2014). Despite evidence that McGurk susceptibility may not be a valid measure of audiovisual integration (Van Engen et al., 2017), others found an increased McGurk susceptibility to be related to an audiovisual benefit for sentences (Grant and Seitz, 1998) and to better audiovisual speech in noise perception (Gieseler et al., 2020). Our results showed a positive correlation at trend level between McGurk illusion and audiovisual speech in noise intelligibility, indicating better audiovisual speech perception with more McGurk illusions. This correlation was present at baseline (no hearing aid experience in participants), but not in the re-test assessment (when half of the participants had hearing aid experience for six months). Hence, it seems that in elderly untreated hearing-impaired participants, an increased McGurk illusion is associated with better audiovisual speech perception. Interestingly, we found that an increase in McGurk illusion after hearing aid fitting was related to a decreased improvement in auditory speech in noise intelligibility when tested in an aided compared with unaided condition. This may suggest, that participants were still influenced by the visual input which may have led to an increased McGurk illusion perception. That in turn seems to inhibit the beneficial effect of the hearing aid when tested in auditory only conditions. Thus, our results suggest that changes in McGurk susceptibility may be relevant for everyday life speech comprehension and communication.

### Functional resting state changes associated with hearing aid use

To assess changes in resting state functional connectivity of the auditory cortex with hearing aid use, we computed an interaction analysis between group (treatment/control) × measurement time point (baseline/after six months). We hypothesized a decrease in functional coupling of the auditory cortex to visual brain regions after hearing aid use of six months (Shiell et al., 2015; Kral et al., 2016; Chen et al., 2017; Puschmann et al., 2019). This hypothesis was confirmed by our ROI-to-ROI analysis that showed decreased auditory cortex coupling with the fusiform gyrus in the treatment group. Moreover, we expected a change in number of perceived McGurk illusions and that this would be associated with changes of functional connectivity between auditory cortex, the fusiform face area and M1 lip area (Kanwisher et al., 1997; Kanwisher and Yovel, 2006; Murakami et al., 2018; Rosemann et al., 2020; Schulte et al., 2020). While we found no significant changes in resting state functional connectivity between the auditory cortex and M1 lip area, the change in connectivity between the auditory cortex and fusiform gyrus was significantly different between treatment and control group. The treatment group showed a significant decrease in connectivity, while the control group showed no significant change (which led to a significant interaction of group × measurement time point). Furthermore, this change was related to the perception of the McGurk illusion in the treatment group.

Surprisingly, the decrease in functional coupling was linked to an increased number of perceived McGurk illusions after six months of hearing aid use in the treatment group. In other words, we found that hearing aid use was associated with (1) a decrease in resting state functional connectivity between auditory and fusiform brain regions, and (2) that this decrease in connectivity was linked to an increase in McGurk illusions. A recent study demonstrated that hard-of-hearing participants compared with normal-hearing participants showed an increased task-modulated functional connectivity between auditory and visual cortex for McGurk as compared with congruent audiovisual stimuli (Rosemann et al., 2020). Other research has also provided evidence for an increased auditory cortex coupling in hard-of-hearing individuals as a sign of cross-modal plasticity (Shiell et al., 2015; Kral et al., 2016; Chen et al., 2017; Puschmann et al., 2019). In a previous study investigating the relationship between resting state functional connectivity and McGurk illusion rate, decreased coupling between auditory and motor regions associated with an increased McGurk illusion rate was demonstrated (Schulte et al., 2020). Hence, it seems that the perception of the McGurk illusion is contrarily associated with functional connectivity between the auditory and motor cortex during the actual McGurk task and between the auditory cortex and fusiform gyrus at resting state. While an increased McGurk illusion rate was found to be associated with an increased functional connectivity between the auditory and visual cortex in hard-of-hearing participants, this increased McGurk illusion response was correlated with a decreased resting state connectivity between the auditory and motor regions. Our study provides evidence that hearing aid use leads to a decrease in resting state functional connectivity between the auditory cortex and fusiform gyrus, most likely indicating less focus on visual input and more reliance on auditory input. However, this decrease was related to an increase in McGurk illusion perception after six months of hearing aid use which was rather surprising. A possible explanation might be that the treatment group was tested without their hearing aids (as mentioned above, The effect of hearing aid use on the McGurk illusion). It is probable that these participants may have learnt to rely more on the auditory input because of wearing the hearing aid in everyday life situations (leading to a decrease in resting state functional connectivity between auditory and visual cortex). However, when being tested without the hearing aids, they still may have paid more attention to the visual input which might have led to an increase in McGurk illusion perception.

### Crossmodal plasticity

Crossmodal plasticity in the auditory cortex has mainly been described in deafness (Lambertz et al., 2005), but also in subjects with slight-to-moderate age-related hearing loss (Campbell and Sharma, 2013, 2014). It was further shown, that neural coupling of the auditory cortex to visual areas is increased as well (Kral et al., 2016; Chen et al., 2017; Puschmann et al., 2019). However, coupling of auditory and visual cortex was found to be decreased with longer durations of hearing aid use in early-deaf individuals leading to the assumption that long-term hearing aid use (of several years) might inhibit crossmodal reorganization (Shiell et al., 2015). Other research in cochlear implant patients (Giraud and Lee, 2007; Lee et al., 2007; Sandmann et al., 2012) and hard-of-hearing individuals (Campbell and Sharma, 2014) has shown a negative relation between crossmodal plasticity in the auditory cortex and speech perception outcome. Hence, the visual takeover of the auditory cortex has been described as maladaptive and it has been suggested that an incomplete reversal of this crossmodal plasticity might interfere with the clinical benefit of a cochlear implant or a hearing aid (Sandmann et al., 2012). However, crossmodal plasticity in the visual cortex has been shown to benefit speech perception outcome after cochlear implantation (Giraud et al., 2001a,b; Chen et al., 2016). Similarly, an increased crossmodal plasticity in the auditory cortex was also found to be correlated with audiovisual integration strength in cochlear implant users (Stropahl and Debener, 2017). Hence, there seems to be adaptive and maladaptive plasticity when it comes to speech perception outcome in cochlear implant patients. Unfortunately, studies assessing crossmodal plasticity before and after hearing aid fitting in mild to severe age-related hearing loss are limited. A recent electroencephalography study showed a reversal of auditory cross-modal reorganization after six months of hearing aid treatment (Glick and Sharma, 2020). They further showed that latencies of the right auditory cortex before hearing aid fitting predicted auditory speech perception outcomes six months after hearing aid use (more crossmodal plasticity predicted worse speech perception). A recent resting state functional connectivity study provided evidence of an increased connectivity between right Heschl’s gyrus and frontal cortex that also correlated with executive function improvement six months after hearing aid use (Ponticorvo et al., 2021). However, neither was the hypoperfusion of the Heschl’s gyrus that was found before the hearing aid fitting reversed, nor did speech perception improve in the follow-up measurement of the treatment group. Thus, it is currently not clear, how much sensory loss (degree and length of hearing loss) induces crossmodal plasticity in age-related hearing loss and whether these changes predict good or bad hearing aid benefit for speech perception (Stropahl et al., 2017).

Moreover, crossmodal plasticity has not only been shown in terms of neural activity in the auditory cortex, but there are several studies assessing functional connectivity of the auditory cortex during different tasks (Kral et al., 2016; Chen et al., 2017; Rosemann and Thiel, 2018; Puschmann et al., 2019) or resting state (Schmidt et al., 2013; Husain et al., 2014; Chen et al., 2018; Puschmann et al., 2019; Rosemann and Thiel, 2019; Schulte et al., 2020). Resting state functional connectivity measures temporal correlations of spontaneous activity between brain regions that are organized into coherent networks (Husain et al., 2014). So-called resting state networks such as the default mode, the dorsal attention and the salience network have been found to be reliably detectable and consistently reproducible by previous research and thus have gained attention in a variety of disorders (Smitha et al., 2017; Yang et al., 2020). In contrast to task-based functional neuroimaging, the benefits of resting state measurements are the short acquisition times (∼10 min) and that results are not confounded by performances in the task. Important for clinical research is that some patient populations such as stroke patients or infants may not be able to perform certain tasks and hence task-based fMRI is not applicable in those patient groups (Zhang et al., 2019; Canario et al., 2021). Further, resting state fMRI allows assessments of functional coupling between specific regions, resting state networks and the whole brain whereas task-based fMRI is limited to neural activity and functional connectivity of brain regions elicited by the particular task (Canario et al., 2021). In our study, we were able to show that functional coupling of the auditory cortex to visual cortex is altered after six months of hearing aid use suggesting more widespread changes in underlying network dynamics (that not only affect the auditory cortex during auditory processing).

### Limitations

It seems that hearing aid use in age-related hearing loss is associated with observable neural changes in the auditory cortex that are linked to an increased McGurk illusion response already after a relatively short period of six months. However, we need to point out some limitations of the current study. On the behavioral level, some of the behavioral results were only significant at trend level (for instance increase in McGurk illusions over time, or the relation between McGurk illusions and audiovisual speech in noise intelligibility). This may be because of the small sample sizes and the large variability in the McGurk illusion. A complicating factor that may also serve as an explanation for that result is the fact that the McGurk illusion in the treatment group was only tested in an unaided condition. We chose to measure the treatment group in an unaided condition to enable comparability between groups and to assess potential general changes that are not associated with specific hearing aid settings. Although the level of stimulus presentation was individually adapted for all participants, we cannot exclude that this created a rather unusual and difficult listening situation, in which the auditory input was not perceived as reliable as with the hearing aids. Because of time limits, we were unfortunately not able to conduct both aided and unaided assessments. Regarding the resting state connectivity data, we only observed a significant change in resting-state connectivity in our ROI-to-ROI analysis and not in the whole-brain analysis. A possible explanation might be lack of power because of the small sample size. Hence, the interaction between group and measurement time point was shown to be significant in the ROI-to-ROI analysis but might not have survived the correction for multiple comparisons in the whole-brain analysis. Thus, this study can only be seen as a pilot study. However, the results may trigger future research on the influence of hearing aid fitting in age-related hearing loss. How long-term use of a hearing aid (more than one year) influences cross-modal plasticity in mild to moderate hearing loss and how this relates to speech perception outcomes should be investigated by future research. Further, future studies should consider assessing audiovisual integration in aided and unaided conditions in hearing aid users as well as larger group sizes.

In conclusion, this is the first study to provide pilot data on the effect of hearing aid fitting on the McGurk illusion and resting state functional connectivity. We demonstrated that hearing aid use is associated with changes in resting state functional connectivity between the auditory cortex and fusiform gyrus, which is also related to the increased number of perceived McGurk illusions. We also found a relation between increased McGurk illusion and decreased hearing aid benefit in auditory speech in noise intelligibility. Our study therefore offers valuable insights into alterations in resting state functional connectivity and changes in audiovisual integration (susceptibility to the McGurk illusion) and speech perception after six months of hearing aid use.

## Synthesis

Reviewing Editor: Christine Portfors, Washington State University Decisions are customarily a result of the Reviewing Editor and the peer reviewers coming together and discussing their recommendations until a consensus is reached. When revisions are invited, a fact-based synthesis statement explaining their decision and outlining what is needed to prepare a revision will be listed below. The following reviewer(s) agreed to reveal their identity: Nathalie Giroud.

The authors have answered all the previous concerns and comments.
